# Confinement Situation of the Spanish Population during the Health Crisis of COVID-19: Resilience Mediation Process in the Relationship of Dispositional Optimism and Psychological Well-Being

**DOI:** 10.3390/ijerph18126190

**Published:** 2021-06-08

**Authors:** Antonio Zayas, Ana Merchán-Clavellino, José Antonio López-Sánchez, Rocío Guil

**Affiliations:** 1 Departamento de Psicología, Instituto Universitario de Investigación para el Desarrollo Social y Sostenible (INDESS), Campus Universitario de Jerez, Avda. de la Universidad s/n., Universidad de Cádiz, 11405 Cádiz, Spain; antonio.zayas@uca.es (A.Z.); rocio.guil@uca.es (R.G.); 2 Departamento de Historia, Geografía y Filosofía, Instituto Universitario de Investigación para el Desarrollo Social y Sostenible (INDESS), Campus Universitario de Jerez, Avda. de la Universidad s/n., Universidad de Cádiz, 11405 Cádiz, Spain; joseantonio.lopez@uca.es

**Keywords:** dispositional optimism, resilience, psychological wellbeing, COVID-19, confinement

## Abstract

The pandemic generated by COVID-19 is one of the most complex challenges humanity has faced in recent years. This study aims to explore the levels of dispositional optimism, resilience and psychological well-being in the sociodemographic and economic situation produced during the state of alarm and to investigate the resilience mediation between optimism and psychological well-being. The sample included 566 volunteers from Spain (73.5% women; *M* = 40.2 years, *SD* = 12.8). An ad hoc questionnaire was applied to request socioeconomic data and dispositional optimism (LOT-R). Resilience and psychological well-being were, respectively, evaluated by the Ryff scale and the Wagnild and Young scale. The results show that older and people with higher educational levels are more optimistic and have better psychological well-being. Well-being is also greater in married, divorced and widowed people and in those who have lived in outdoor spaces. However, those with spaced housing were more optimistic. Finally, it was found that the most optimistic people have better psychological well-being and that this is increased by the mediation process exercised by the ability to overcome adversity, provided age and educational level are controlled. It can be concluded that the design of preventive programs focused on improving strengths, positive emotions and skills in the population would be convenient to protect mental health.

## 1. Introduction

The pandemic generated by SARS-CoV-2 and the associated disease, COVID-19, is one of the most complex challenges that humanity has faced in recent years. The beginnings of this pandemic date back to December 2019, where several cases of viral pneumonia were detected in China (Wuhan) [1]. The World Health Organization (WHO) [1] pointed out the origin of the disease in a new coronavirus called severe acute respiratory syndrome coronavirus 2 (SARS-CoV-2), but it was not until 30 January 2020, when the WHO [1] declared the disease as a “public health emergency of international concern,” and on 11 February 2020, this new disease was named “COVID-19,” and on 11 March, it was declared a global pandemic, affecting more than 100 countries.

In Spain, from the time the first COVID-19 fatality was reported on 13 February to 14 March [2,3], there was a rapid spread of the infection and an increase in deaths caused by the disease. To control the spread of this virus throughout the country, the Spanish government declared a state of alarm that ended on 21 June 2020.

This state of alarm implied a confinement situation for the whole Spanish territory. Home isolation was imposed, with very strict limitations on the freedom of movement of citizens, including the prohibition of non-essential commercial, educational, cultural, etc. activities, allowing only mobility to buy basic products (food and medicines), to attend to health centers, or for working or caring dependents [3].

Therefore, the Spanish population began to experience unpredictable changes at an unprecedented speed. On the one hand, the population faced a sudden and unpredictable situation, such as a pandemic, a crisis that produces negative psychological effects on the population [4,5,6,7,8]. Studies show that this health crisis is producing, in the general population, several feelings and emotions such as uncertainty, fear, distress, and vulnerability that are related to helplessness, fragility, insecurity due to the possibility of dying from infection [6]. However, on the other hand, due to the measures that were taken to stop the infections, the population has remained confined at home, producing changes in their family, social, and work dynamics.

In fact, due to these restrictions, many people have lost their jobs immediately, temporarily, or permanently, producing an increase in job insecurity during the state of alarm [9]. In this vein, studies show how experiencing this financial stress is associated with poorer mental health [10]. Job uncertainty generates levels of insecurity [10,11,12] that are significantly associated with psychological well-being [13]. Additionally, having lower levels of income is associated with lower levels of subjective well-being [14,15,16]. It has already been observed that during the SARS-CoV-2 crisis, people with monthly incomes below 800 euros have reported greater emotional consequences and psychiatric symptoms [11]. Another socio-economic aspect of interest in the state of alarm and, therefore, in the restriction of rights, is the type of housing. Specific studies find that there is a relationship between the type of home and its conditions with health and well-being [11,17].

Resilience and optimism are variables that have been widely studied from positive psychology because this approach focuses on the strengths or specific attributes that people have to face adverse situations in the most adaptive way possible [18,19]. Dispositional optimism is a cognitive concept (expectations for future results), understood in a continuum of opposite poles, in which on one side are people who have positive beliefs and look at the world in a positive way and on the other those who have a pessimistic view of life [20,21]. A process that also involves emotional and motivating components, in fact, that optimistic force or impulse produces more perverse, successful, and general well-being oriented people [22,23,24]. Resilience is a dynamic process between the individual and an unfavorable context, in which there is a real and significant threat to their health, well-being, development processes, or mental health, in which the person uses both intrinsic and extrinsic resources to quickly establish a physical, psychological, and social balance with leads to healthy life [25,26]. Some dimensions that are considered in people with resilience are equanimity, perseverance, self-confidence, personal satisfaction, and feeling good [27]. However, research has confirmed other psychological traits shared by this type of people, such as being optimistic people and having hope in life [28,29].

It has been shown that both concepts positively correlate with psychological well-being, satisfaction with life, and positive emotions [30,31,32,33,34], but negative correlations have been found with respect to negative emotions, anxiety, depression, etc. [29,33]. Some studies confirm that resilience has a mediating role in the relationship between dispositional optimism and psychological well-being in certain traumatic or stressful situations [28]. Therefore, the present study aimed to explore this relationship during the state of alarm due to the SARS-CoV-2 pandemic, which, as mentioned above, results in crisis and stressful situations, both due to health aspects and restrictions of rights. Specifically, a greater impact of emotional distress and worse mental health has been found in people who had monthly incomes lower than 800 euros, women, people aged 18–26 years, with lower income levels, lower levels of education, the unemployed, and people with reduced space in their homes [6,11,35,36,37]. Additionally, positive relationships were associated with resilience in older people, men, those with university studies, and people without a partner. Finally, it has been detected that optimism predicts high levels of resilience [38], which in turn dampens the negative effects of the pandemic [5,39].

Therefore, this study aims to compare the levels of dispositional optimism, resilience, and psychological well-being according to sociodemographic data such as sex, marital status, and educational level. In addition, the study aims to compare according to the socio-economic situation of Spaniards during the pandemic situation with a non-essential mobility restriction, where factors such as employment, the level of family income, the number of members with whom they live, the habitable space, and the availability of air fresh are prominent variables. We expect that job losses, lower economic income, and having been confined to fewer square meters and with more people will be associated with worse indicators of mental health, such as lower levels of optimism, resilience, and well-being. A secondary objective is to evaluate the mediation process that maintains resilience in the relationship of dispositional optimism with psychological well-being but controlling all those sociodemographic and economic variables that have characterized this situation. It is hypothesized that the most optimistic people will have a better state of psychological well-being and that this is increased by the mediation process exercised by the ability to overcome adversity in such a way that the most optimistic people will be more resilient and report better psychological well-being.

## 2. Materials and Methods

### 2.1. Study Design and Participants

This is a cross-sectional study. The recruitment was carried out through social networks in the Spanish population of legal age (from 18 years old). The sample was selected using non-probabilistic methods. A total of 566 people participated.

The data was collected during the period of the state of alarm established by the Spanish Government from March 14 to June 21, 2020. All participants voluntarily completed the self-administered online questionnaires. They previously were informed of the objectives of the study and notified that they could terminate their participation at any time if they wished. Participation in this study was, thus, anonymous and confidential. The study was conducted in compliance with the 1975 Declaration of Helsinki, and all participants signed the informed consent form.

### 2.2. Measures

We used the Wagnild and Young Resilience Scale [40]. This scale consists of 25 items on a 7-point Likert-type scale, where 1 means disagrees, and 7 means strongly agrees. Thus, the higher the score, the higher the resilience, with scores varying between 25 and 175 points. This questionnaire measures resilience understood from two factors such as personal competence and acceptance of oneself and life. The resilience characteristics are equanimity, perseverance, self-confidence, personal satisfaction, and feeling good on their own. In our study, the total scale has an adequate internal consistency with a Cronbach’s alpha of 0.903. The model with 5 first-order factors and 1 second-order factor (resilience) shows indicators of good fit to the model *χ*^2^ = 14.721, *p* = 0.005; CFI = 0.992; TLI = 0.980; RMSEA = 0.069; SRMR = 0.016.

We also used the Life Orientation Test-Revised (LOT-R). A Spanish version of this test was administered, which measures optimism–pessimism factors [41]. It consists of ten items: 3 items refer to optimism and three to pessimism, and 4 items intend to avoid detection of the objective of the questionnaire. Each item ranges from 0 to 4 (0 corresponding to very much in disagreement, and 4 corresponding to very much in agreement). To obtain the total score, the pessimistic items (3, 7, and 9) have inverted values, and the values of the 6 inverted optimism and pessimism items must be added together. Reliability analysis for the optimism score reveals a Cronbach’s alpha of 0.737. The model with a single factor (optimism) shows the following indicators of fit to the model *χ*^2^ = 102.659, *p* = 0.000; CFI = 0.90; TLI = 0.80; RMSEA = 0.145; SRMR = 0.05.

A third scale used was the Ryff’s Psychological Well-Being Scale (1989) [42], adapted by Van Dierendonck [43] and translated into Spanish by Díaz, Rodríguez, Blanco, Moreno, Gallardo, Valle, and van Dierendonck, [44]. This has 39 items, with a Likert-type response format ranging from 1 to 6, in which 1 = Strongly disagree and 6 = Strongly agree. The measured construct can be divided into the following sub-dimensions; Self-acceptance, Positive relationships, Autonomy, Mastery of the environment, Purpose in Life, and Personal growth. However, in our study, we will consider the total score as a measure of psychological well-being. The internal consistency of the total scale is 0.918. The 6-factor model and a second-order factor (psychological well-being) show indicators of good fit to the proposed model *χ*^2^ = 40.091, *p* = 0.000; CFI = 0.973; TLI = 0.949; RMSEA = 0.084; SRMR = 0.027.

An ad hoc questionnaire was also included containing questions on socio-demographic variables (gender, educational level, marital status, autonomous community where confinement took place), on economic data before the health crisis (employment situation and monthly income levels) and during confinement, and considering if there were negative changes in working conditions and income. For the latter, it was established the following question: How has the COVID-19 crisis affected income in your home? The allowed answer was that it did not influence or, on the contrary, the pandemic worsened income, including the resignation of some income, or the main source of income disappeared. To measure the change in working conditions, participants were asked about the employment situation after the start of the state of alarm, with the following answers: the situation did not change or changed negatively, including in this category the following answer options: unemployed, reduction of working hours and working conditions, and working activity did cease or decrease. The last question was about the type of housing in which they were confined (meters of the housing, outdoor space available, such as a terrace, patio, etc., and the number of people sharing the house during this period).

### 2.3. Statistical Analysis

Basic descriptive statistics (percentages, means and standard deviations) of the study variables were analyzed. Chi-square analyses were performed to observe the association between the nominal and categorical variables of the study. For the scale variables, since the principles of normality were not met, different nonparametric analyses were performed for two (Mann–Whitney U test) and for independent samples (Kruskal–Wallis H test), for testing if there were significant differences in resilience, dispositional optimism and psychological well-being for certain economic and socio-demographic variables during the alarm state period. Analyses were established according to gender, level of education, marital status, changes in employment, and income levels, and finally, compared according to housing, particularly the number of people sharing the house, the meters of the dwellings, and the availability of outdoor space. Likewise, correlations were performed with Spearman’s Rho test between the main variables of the model and age.

Finally, to evaluate the reliability and validity of the scales, Cronbach’s alpha coefficient and the Confirmatory Factor Analysis (CFA) were calculated. In this last analysis, the maximum likelihood estimator was used, and the indices show a good fit when the Comparative Fit Index (CFI) and the Tucker–Lewis Index (TLI) have values ≥ 0.90, the Mean Square Error approximation values (RMSEA) ≤ 0.05 or 0.08 and standardized values of residual mean square root (SRMR) ≤ 0.05.

**Figure 1:**
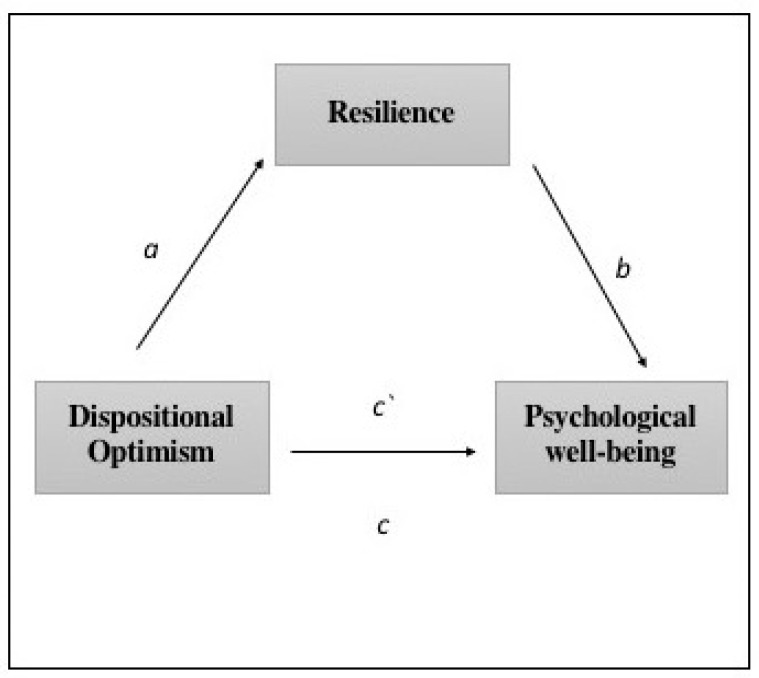
The conceptual and statistical scheme of the resilience mediation between dispositional optimism and psychological well-being (parameter (c’) is the direct effect of Dispositional Optimism (X) on Psychological well-being (Y) controlling for the mediating variable, (a) is the direct effect of X on M, (b) is the direct effect of M on Y, and the total effect (c) is the sum of the direct and indirect effects, the in which mediator is excluded from the regression).

The SPSS 25.0 statistical package (IBM Corp, Armonk, NY, USA) was used, and through the macro Process [45], the mediation analysis was established with a 95% confidence interval and a number of bootstrapping samples of 10,000. The estimations of each analysis were performed through respective unstandardized regression coefficients (coeff), standard errors (SE), t-values and significance levels (*p*), as well as by the different values of the lower limit (LLCI) and upper limit (ULCI) of the confidence interval. The interpretation of significance was performed using the values of each lower and upper limit of the confidence interval, in which the number zero between this interval confirmed no significant results. The simple mediation analysis was performed using model 4, analyzing whether the effect of the independent variable (X) (dispositional optimism) on the dependent variable (Y) (psychological well-being) may be mediated by the mediating variable (M) (resilience), including as covariates the economic and sociodemographic variables related to the situation of confinement. As shown in Figure 1, parameter (c’) is the direct effect of X on Y controlling for the mediating variable, (a) is the direct effect of X on M, (b) is the direct effect of M on Y, the indirect effect (ab) is the effect by the mediating variable, and the total effect (c) is the sum of the direct and indirect effects, the in which mediator is excluded from the regression.

## 3. Results

### 3.1. Description of the Sample for Economic and Socio-Demographic Data before the State of Alarm

The sample included 566 Spaniards, distributed throughout 15 autonomous communities of the Spanish geography: 86.7% are from Andalusia, 4.8% from the Community of Madrid, 2.7% from Catalonia, 1.4% from the Canary Islands, 1.1% from Castile and La Mancha, 0.9% from the Balearic Islands, 0.5% from Castile and Leon, 0.4% from the Community of Valencia, Extremadura, and the Basque Country, and 0.2% from Asturias, Ceuta, Galicia, Murcia, and Navarre.

Table 1 shows the socio-demographic and economic data before the state of alarm in Spain for the total sample n total, 26.5% of men and 73.5% of women participated. The average age of the participants is 40.2 years (ST = 12.8).

### 3.2. Sociodemographic and Economic Indicators of Confinement and Their Relationship with Dispositional Optimism, Resilience, and Psychological Well-Being

During the state of alarm, 29.7% of the population reduced their working conditions, and 40.6% of the participants stated that their income had been reduced during the state alarm, with significant partial or totals. The results show that there are statistically significant differences in the working conditions between the previous work situation and during the state of alarm (χ^2^ (8) = 131.44, *p* = 0.000, V = 0.482), and also significant differences regarding family income (χ^2^ (8) = 74.25, *p* = 0.000, V = 0.362). Thus, the employment situation was reduced in self-employed (with hired personnel, *p* = 0.000 and without hired personnel, *p* = 0.000) and hired in the general regime (temporary, *p* = 0.000 as indefinite, *p* = 0.011) regarding other conditions. However, income has decreased to a greater extent in self-employed (with contracted personnel, *p* = 0.001 and without contracted personnel, *p* = 0.000). Regarding income levels before the crisis, we did not find a significant change in working conditions (χ^2^ (5) = 8.52, *p* = 0.130, V = 0.123) or the economic level (χ^2^ (5) = 9.17, *p* = 0.103, V = 0.127), that is, the effects of the crisis at the economic level have been homogeneous among people with different types of salary.

Regarding the type of housing during the state of alarm, 73.1% of Spanish people have been confined to houses with a range of square meters between 60 and 150. It should be noted that 68.7% reported outdoor space availability during this crisis (terraces, patios, gardens, etc.), and there is a significant association with respect to the type of dwellings with outdoor spaces availability (χ^2^ (4) = 66.21, *p* = 0.000, V = 0.342), being the houses bigger than 90 square meters (between 90–120 square meters, *p* = 0.023, between 120–150 square meters, *p* = 0.000 and more than 150 square meters, *p* = 0.000) those with greater availability of terraces, gardens, or patios, compared to those smaller than 90 square meters. Additionally, 9.7% of participants were living alone during the confinement, while 12.9% lived with more than three people.

The general trend indicates a reduction in incomes, living with 1–3 people, in houses ranging from 60–90 m, and with some outside spaces. Table 2 depicts the descriptive and statistical analyses conducted to establish the differences that the population has reported in optimism, resilience, and psychological well-being, according to the different sociodemographic and economic indicators generated by this confinement situation.

Resilience showed significant differences regarding marital status, in such a way that single people have less resilience capacity than those who live or have lived with someone. The level of education shows differences between the groups for both optimism and the level of psychological well-being. In the pairwise comparisons, it is observed that people without primary studies or without any studies show lower levels of optimism compared to people with university studies (Z = −2.29; *p* = 0.022), postgraduate (Z = −3.12; *p* = 0.002) and doctoral studies (Z = −2.35; *p* = 0.019). Regarding psychological well-being, differences were found between the following groups: no primary studies/doctorate studies (Z = −2.81; *p* = 0.005); no studies/primary–postgraduate studies (Z = −1.99; *p* = 0.047); secondary studies–doctorate (Z = −2.23; *p* = 0.026); university studies–doctorate (Z = −2.78; *p* = 0.005), showing better well-being in those people with a higher level of studies or academic qualifications. The optimism variable also showed significant differences regarding the type of housing during the lockdown. Thus, for example, people who lived in houses between 60–90 m were more optimistic than those who lived in houses with fewer meters (<60) (Z = −2.24; *p* = 0.025) and those who lived in houses of 120–150 m (Z = −2.46; *p* = 0.014).

Finally, age shows positive correlations with resilience, dispositional optimism, and psychological well-being (see Table 3), although the effect size of the relationships is not very high (<0.3). However, there are strong positive correlations between the three psychological variables; that is, people with greater resilience are more optimistic and have better psychological well-being, and well-being is higher for those who are more optimistic.

### 3.3. Analysis of the Resilence Mediation between Optimism and Psychological Well-Being, Controlling for Economic and Sociodemographic Variables

Table 4 shows the simple mediation analysis of resilience in the relationship between dispositional optimism and psychological well-being, including all those economic and sociodemographic factors that have shown some relationship with the variables of our model as covariates. The results yielded significant values for direct and total effects, as well as for indirect effects, considering that 0 value is not included in the 95% confidence interval [0.9157/1.079]. In the first regression, it is shown that dispositional optimism explains the 27.1% of the resilience variance (R^2^ = 0.271, F = 34.62, *p* = 0.000), with no significant covariates (*p* > 0.05). Regarding psychological well-being, the optimism factor represented the 44.5% of the explained variance (R^2^ = 0.445, F = 74.79, *p* = 0.000), without significant covariates (*p* > 0.05). However, the indirect effects analyzed were also significant; therefore, psychological well-being showed an explained variance of 63.8% according to the global model, that is, resilience is included as a mediating variable (R^2^ = 0.638, F = 140.38, *p* = 0.000) and with the following significant covariates: age, *b* = −0.177, 95% CI [−0.303; −0.050] and marital status, *b* = 3.301, 95% CI [0.098; 6.504].

## 4. Discussion

The first objective of this study was to compare the different levels of dispositional optimism, resilience, and psychological well-being, according to the sociodemographic and economic data of Spaniards during a pandemic situation with non-essential mobility restrictions.

Regarding the sociodemographic and economic data, 28.5% of the sample has an indefinite employment situation, with a 15.1% being public officials, although 16.8% has temporal employment, and 27.4% are unemployed. These data were in line with Spanish statistics, which reveal that unemployment in Spain in February 2020 was 13.9%, and in Andalusia, the Spanish region where the majority of our sample is from, unemployment increased to 21.3%. Even so, 86.7% of the sample reports a family monthly income before COVID-19 superior to thousand euros, with 45.2% of salaries being above 2000 euros. The average salary in Spain in 2019 stood at 27,537 euros per year, that is, levels above 2000 euros per month [46]. Regarding the employment situation during the state of alarm, our results highlight that for 29.7% of the sample, there were changes in the working conditions, reporting worst conditions, and in 40.6% of the participants, the income was reduced compared to the pre-pandemic situation. These data are in line with other studies, showing that measures as social distancing measures and the Government order to stay at home to contain the COVID-19 pandemic affect economic activity in Spain [9]. Apparently, this situation has generated job insecurity during the state of alarm [10]. Finally, 28.6% of the dwellings where our sample was during confinement corresponded to houses with less than 60 m, followed by dwellings between 90–120 m (26.0%), with 68.7% of the houses having outdoor spaces and 28.0% of the sample coexisting with more than three members and 27.7%, alone. These results confirm that this new and unexpected situation caused a change in lifestyle and a negative socio-economic effect on the population.

To begin with the analysis of the first objective, we found that younger people had more psychological consequences, in terms of lower levels of optimism, resilience, and psychological well-being, which reveals that age acts as a protective factor in the socio-sanitary crisis caused by the pandemic, in the sense that this crisis apparently had a minor psychological impact on older people. Therefore, Ozamiz-Etxebarria, Idoiaga-Mondragón, Dosil-Santamaría, and Picaza-Gorrotxategi (2020) [47] found that symptoms of depression, anxiety, and stress were more frequent in young people, just as young people were less resilient [37]. This last study [37] also found higher levels of resilience in singles, although our data showed higher levels in married, widowed, or divorced people. Our results also show that singles have higher levels of psychological well-being. Future studies should elucidate if the group of married, divorced, or widowed could be in a larger family group and thus have greater social support, which might be a factor that improves the adaptive behaviors to cope with the situation and cushion the adverse effects of the pandemic. Another sociodemographic data that other studies confirm is the relationship between psychological impact and educational level [5,11,36]. Our data reveals that people with a higher degree of studies have better levels of dispositional optimism and psychological well-being. Although this alarm situation seems to produce labor and economic changes in the Spanish population, our results are not in line with the association between the existence of job insecurity and poorer psychological well-being [13]. This can be explained by the fact that there may be other protective factors, such as the wages that companies have granted to their employees, the Government economic aids to alleviate the economic crisis, the savings they may have, and other regulatory mechanisms to protect employees. Regarding the socioeconomic variables, we found that people having outdoor spaces reported higher levels of psychological well-being, and having more square meters in homes seems to be associated with better levels of dispositional optimism. Therefore, people with more square meters and open spaces to live have greater mental health protection [11], although some studies suggest that the key for this could be the feeling of comfort in homes [6].

In short, the living conditions that people have in the current health crisis are confirmed by other studies about confinement or crises, all this indicating that the fear of the unknown and the uncertainty generated by the situation can lead to mental disorders [7,8,48]. These mental disorders could be reduced via appropriate and adaptive coping skills for each specific situation. Our second objective was to know the mediation process that resilience has in the relationship between dispositional optimism and psychological well-being, controlling the sociodemographic and economic variables, that are, characteristics of the situation. The results reveal significant differences in some of the evaluated concepts. It was hypothesized and confirmed that the most optimistic people have a better state of psychological well-being, which is increased by the mediation process exercised by the ability to overcome adversity in such a way that the most optimistic people are more resilient and the most resilient, the better psychological well-being.

These findings show the importance of the psychosocial approach to increase the population’s strengths or specific attributes to face adverse situations in the most adaptive way possible. Therefore, it can be argued that to reduce the impact of the pandemic on psychological well-being, positive expectations for the future should be increased. Some studies have shown the possibility of increasing optimism, albeit temporarily, since it will help the person to put into action a mechanism towards healthy behaviors through its motivating and emotional effect [20,21,22,23,24]. Furthermore, promoting resilient skills seems to be more effective and long-lasting for improving psychological well-being and reducing adverse mental effects [49,50], even during this pandemic situation [51]. Therefore, more optimal results may be obtained in the prevention of mental health if a complementary program is implemented, which may help people to have a more optimistic vision and promote resilient behaviors in the short and long term. The design of such a program should homogenize the target population in terms of age and educational level since they are two factors with significant effects in our global mediation model.

Some limitations of this study must be considered. The sample had an unequal demographic distribution since 86.7% of the study population was Andalusian, and 73.5% were women. Therefore, it may be an under-representation of some regions. Moreover, the difficulty to control social desirability effects when using self-administered scales is known, although an attempt to reduce these effects has been described through anonymity and preventing that the participants know the evaluated term.

Despite this, future studies should include bigger samples, taking into account the geographical location and the restriction measures in each autonomous community or region. Additionally, it would be interesting to evaluate these long-term relationships since our recordings were focused on the first moments of the pandemic and under the most restrictive measures. Finally, it would be relevant to incorporate measurements of social support as a variable of interest, which could increase people’s adaptive skills to face this type of situation.

## 5. Conclusions

This research provides, in the middle of a pandemic crisis, knowledge about those relevant variables that are associated with mental health indicators. The findings reveal that certain specific attributes, such as having a greater positive vision of the world and resilient behaviors, may improve some mental health indicators, such as psychological well-being. Therefore, these results promote the design of preventive programs focused on improving personal strengths, positive emotions, and skills in the population as a measure to protect mental health.

## Figures and Tables

**Table 1:** Description of the sample for economic and socio-demographic data before the state of alarm.

Socio-Demographic Variables	*n*	*%*	Economic Variables	*n*	*%*
Gender			Employment situation		
Woman	416	73.5	Unemployment	154	27.4
Man	150	26.5	Freelance with hired staff	14	2.49
Marital Status			Freelance without hired staff	25	4.45
Single	266	47	Hired in general temporary regime	94	16.73
De facto couple	35	6.18	Hired in an indefinite general regime	160	28.47
Married	213	37.63	Official	85	15.12
Widowed	11	1.94	Seasonal self-employed summer season	2	0.36
Divorced	41	7.2	Retirement	28	4.98
Level of studies (completed)			Leave	4	0.71
No studies	1	0.18	Monthly Income level		
Primary Studies	21	3.71	Less than 500 €	19	3.36
Secondary Studies	188	33.2	Between 500–1000 €	56	9.89
University Studies	242	42.76	Between 1000–1500 €	133	23.5
Postgraduate studies	76	13.43	Between 1500–2000 €	102	18.02
Doctorate Program	38	6.71	Between 2000–2500 €	96	16.96
			More than 2500 €	160	28.27

**Table 2:** Descriptive statistics (means and standard deviations) and statistics (Mann–Whitney *U* test/Kruskal–Wallis *H* test) for resilience, dispositional optimism, and psychological well-being for different economic and socio-demographic variables.

			Resilience		Dispositional Optimism		Psychological Well-Being	
Variable	Category	*n*	*M*	*SD*	*M*	*SD*	*M*	*SD*
Gender	Woman	416	138.14	18.16	22.02	4.37	178.52	25.02
	Man	150	138.99	17.93	22.32	4.22	176.39	26.39
	Z		−0.698		−0.553		−0.528	
	*p*		0.485		0.580		0.598	
Marital Status	Unmarried (single and de facto couple)	301	136.72	18.36	21.81	4.50	175.62	26.21
	Marrried/Widowed/Divorced	265	140.22	17.63	22.43	4.10	180.61	24.18
	*Z*		−2.497		−1.361		−2.311	
	*p*		0.013 *		0.173		0.021 *	
Level of studies	No studies/Primary Studies	22	142.18	20.13	20.32	3.39	171.55	22.23
	Secondary Studies	188	137.84	18.79	21.77	4.81	177.61	26.70
	University Studies	242	137.41	18.15	22.12	4.24	176.08	25.85
	Postgraduate studies	76	139.12	16.80	23.08	3.72	182.26	20.99
	Doctorate Program	38	143.26	14.84	22.66	3.57	186.74	23.30
	*H*		4.521		10.004		11.181	
	*p*		0.340		0.040 *		0.025 *	
Change in Employment situation	No	398	138.47	17.94	22.09	4.29	178.15	24.93
	Yes	168	138.10	18.48	22.11	4.44	177.50	26.51
	Z		−0.210		−0.097		−0.155	
	*p*		0.833		0.923		0.877	
Change in income.	No	336	139.15	18.17	22.29	4.31	179.12	24.92
	Yes	230	137.21	17.94	21.82	4.35	176.26	26.01
	Z		−1.071		−1.279		−1.083	
	*p*		0.284		0.201		0.279	
People you live with	Only	55	138.02	17.58	22.53	4.07	173.58	27.70
	1	151	140.40	17.91	22.39	4.43	179.84	27.01
	2	145	138.77	18.95	21.98	4.21	177.94	24.46
	3	142	136.58	16.88	22.11	4.35	179.51	21.92
	>3	73	137.07	19.34	21.40	4.50	174.36	28.01
	*H*		3.979		2.510		3.907	
	*p*		0.409		0.643		0.419	
Housing meters	<60	51	133.63	20.61	21.12	4.67	171.55	28.63
	60–90	200	138.84	16.76	22.07	4.21	177.23	23.33
	90–120	178	139.59	18.27	22.79	3.96	180.24	26.01
	120–150	62	136.18	16.61	20.97	4.86	179.66	23.80
	>150	75	139.20	20.12	22.15	4.54	177.43	27.77
	*H*		6.329		9.506		6.893	
	*p*		0.176		0.050 *		0.142	
Housing having open air	No	177	136.63	18.33	21.64	4.36	174.32	24.42
	Yes	389	139.15	17.94	22.31	4.30	179.61	25.67
	Z		−1.881		−1.901		−2.646	
	*p*		0.060		0.057		0.008 **	

* *p* < 0.05; ** *p* < 0.001.

**Table 3:** The correlations between dispositional optimism, resilience, psychological well-being, and age.

	Correlation		
	1	2	3
1. Age	-		
2. Resilience	0.157 **	-	
3. Dispositional optimism	0.116 **	0.465 **	
4. Psychological well-being	0.102 *	0.683 **	0.652 **

* *p* < 0.05; ** *p* < 0.01.

**Table 4:** Simple mediation analysis of optimism regarding the relationship between resilience and psychological well-being, controlling for economic and socio-demographic variables during confinement in Spain.

				IC 95%	
**Effects**	**Path**	**Coeff**	**SE**	**LLCI**	**ULCI**
Direct between optimism and resilience	*a*	2.101	0.1536	1.799	2.403
Direct between resilience and psychological well-being	*b*	0.721	0.0419	0.639	0.803
Direct between optimism and psychological well-being	*c'*	2.345	0.1757	1.999	2.689
Total effect between optimism and psychological well-being	*c*	3.859	0.1880	3.490	4.229
**Indirect effects**	**Effect**	**SE**	**BootLLCI**	**BootULCI**	
Total indirect effect	1.515	0.156	1.224	1.833	

Abbreviations: Coeff = non-standardized regression coefficients; SE = standard error; IC 95% = confidence interval 95%; LLCI = lower limit; ULCI = upper limit; BootLLCI, bootstrapping lower limit confidence interval; BootULCI, bootstrapping upper limit confidence interval; Covariates: age, marital status, level of education, meters of housing, and house with outdoor space; N = 566.

## Data Availability

The data presented in this study are available on request from the corresponding author. The data are not publicly available due to privacy issues.
